# Relation between resilience and personality traits: The role of hopelessness and age

**DOI:** 10.1111/sjop.12866

**Published:** 2022-09-03

**Authors:** Marta Nieto, María E. Visier, Inmaculada Nayara Silvestre, Beatriz Navarro, Juan Pedro Serrano, Vicente Martínez‐Vizcaíno

**Affiliations:** ^1^ Faculty of Medicine University of Castilla‐La Mancha Albacete Spain; ^2^ Applied Cognitive Psychology Unit University of Castilla‐La Mancha Albacete Spain; ^3^ Faculty of Nursing University of Castilla‐La Mancha Cuenca Spain; ^4^ Health and Social Research Center, University of Castilla‐La Mancha Cuenca Spain; ^5^ Faculty of Medicine Universidad Autónoma de Chile Talca Chile

**Keywords:** Resilience, personality traits, hopelessness, age

## Abstract

Resilience refers to the process by which individuals use the ability to cope with challenges to successfully adapt to adverse situations, inclining towards the future and hope. The main aim of this study was to analyze the relation between resilience, personality traits, and hopelessness. Furthermore, we conducted comparisons between two age groups: young and older adults. The sample comprised 439 Spanish participants (66.7% women; *M* = 43.73, *SD =* 26.41; age range = 18–98 years). The Connor–Davidson Resilience Scale, NEO‐Five Factor Inventory, and Beck Hopelessness Scale were used to measure the main study variables. The results revealed a negative relation between resilience and neuroticism, and a positive association with the other personality traits. Additionally, levels of resilience were found to be negatively related to hopelessness. The group of older adults showed significantly lower resilience levels than the young adults, although age was not a significant predictor of resilience. Neuroticism, extraversion, openness, and hopelessness were the only predictors of resilience for the current study. This work contributes to the study of resilience and related factors, by attempting to understand the role of resilience and resistance to risk and how individuals tackle challenges over time, with important implications for mental health.

## INTRODUCTION

Mental illness is one of the main causes of disability around the world (World Health Organization, 2022). It affects not only the lives of patients but can also have a lasting impact on their immediate environment and society (World Health Organization, 2019). Stressful experiences, such as trauma, life changes, wars, illness, or pandemics have been considered risk factors for mental health (e.g., Bryant, Schnurr & Pedlar, 2022; Harnett, van Rooij, Ely *et al*., 2021; Willey, Mimmack, Gagliardi *et al*., 2022; Zhu, Zhang, Zhou, Li & Yang, 2021). However, it is also worth noting that many people exposed to stressful situations recover quickly or suffer no significant psychological and functional deterioration (Bonanno, Westphal & Mancini, 2011; Kalisch, Baker, Basten *et al*., 2017). There may be many reasons for this, among which resilience has been highlighted as a key trait in achieving good mental health (Färber & Rosendahl, 2020).

Resilience refers to the process by which people use the ability to cope with challenges and new circumstances to positively adapt to adverse contexts, leaning towards the future and hope (Barton, McKay, Garvis & Sappa, 2020; Murphy, 1987). Likewise, it is about a multidimensional construct determined by intrinsic and extrinsic factors that underlie an individual's cognitive, social, emotional, behavioral, and psychological functioning (Malhi, Das, Bell, Mattingly & Mannie, 2019; Masten, 2015). Focusing on the intrinsic approach, resilience can be presented as a trait that moderates the negative impact of stress and facilitates adaptive development (Connor & Davidson, 2003; Ong, Bergeman, Bisconti & Wallace, 2006). Trait‐resilience has been commonly operationalized using the Connor–Davidson scale (CD‐RISC; Connor & Davidson, 2003). More specifically, the CD‐RISC assesses personal characteristics, such as self‐efficacy and optimism, which can be effective in adequately managing stress and enhancing adaptive development (Rodríguez, Alonso & Hernansaiz, 2016).

Resilience is a construct that has been studied in different perspectives (Bonanno et al., 2011; Salisu & Hashim, 2017; Southwick, Bonanno, Masten, Panter‐Brick & Yehuda, 2014). One such perspective focuses on resilience as personality characteristics that manifests itself in response to life circumstances and individual profiles (Cloninger & Zohar, 2011; Connor & Davidson, 2003; Oshio, Kaneko, Nagamine & Nakaya, 2003). These profiles reflect an individual's characteristic thoughts, feelings, and behaviors, namely, personality (Wagner, Lüdtke & Robitzsch, 2019). The Five‐Factor Model is typically taken as the reference when operationalizing personality (Costa & McCrae, 1992). This model comprises five broad categories, namely, neuroticism, extraversion, openness, agreeableness, and conscientiousness. Personality traits tend to remain stable over the lifespan, although they may undergo changes as a result of maturation processes and life experiences. In this sense, it has been suggested that neuroticism and extraversion decrease with age, while agreeableness and conscientiousness increase across the lifespan. Generally, openness increases in adolescence and declines in aging (Costa, McCrae & Löckenhoff, 2019).

A resilient personality is characterized by high levels of extraversion, agreeableness, conscientiousness, openness, and low levels of neuroticism (Alessandri, Vecchione, Donnellan, Eisenberg, Caprara & Cieciuch, 2014). In general, negative relations have been found between resilience and neuroticism, and resilience has been positively associated with the other traits (see Oshio, Taku, Hirano & Saeed, 2018, for a review). Neuroticism and extraversion have been more specifically related to resilience (Lü, Wang, Liu & Zhang, 2014), as these traits can be more significantly affected by life events (Ogle, Rubin & Siegler, 2014; Sarubin, Wolf, Giegling *et al*., 2015). Considering that neuroticism has been associated with adverse psychological outcomes, such as depression and hopelessness (Chioqueta & Stiles, 2005; Grav, Stordal, Romild & Hellzen, 2012; Hjemdal, Friborg & Stiles, 2012; McDonnell & Semkovska, 2020), its negative effect on resilience is unsurprising. Furthermore, it has been suggested that when people are coping with stress and their resilient resources are scarce or non‐existent, hopelessness can be a predictable outcome (Hjemdal *et al*., 2012). In contrast, extraversion leads to more adaptive stress management (Schneider, Rench, Lyons & Riffle, 2012).

Resilience could be the key to explaining resistance to risk and the way people cope with challenges across the lifespan (Färber & Rosendahl, 2020). However, age‐related differences in resilience are not yet conclusive. While some studies have reported that as people age, they become more resilient (Campbell‐Sills, Forde & Stein, 2009; Gillespie, Chaboyer & Wallis, 2009), others have shown a negative relation between resilience and age (Beutel, Glaesmer, Decker, Fischbeck & Brähler, 2009; Lamond, Depp, Allison *et al*., 2008). Even in recent reviews, no significant associations have been found between these variables (Färber & Rosendahl, 2020; Lee, Nam, Kim, Kim, Lee & Lee, 2013). Indeed, it seems that the results are still inconclusive, due mainly to methodological aspects, such as the existence of a wide range of evaluation instruments, covariates, and sample characteristics (Lee *et al*., 2013; McGinnis, 2018; Pulido, Fernández & Lopez, 2020).

## THE CURRENT STUDY

In light of the above, resilience seems to be a significant construct for the promotion of health and prevention of mental illnesses because it could help people recover, maintain, and improve their psychological well‐being when they have been exposed to stressful experiences (Färber & Rosendahl, 2020; Ungar & Theron, 2019). Furthermore, the study of resilient individuals and the mechanisms that protect them is a strategy recommended to combat stress‐related diseases (Kalisch *et al*., 2017). Considering that individuals' responses to stressful situations are the result of a complex interaction of factors, including personal characteristics (Biggs, Brough & Drummond, 2017), this work aims to complement the existing literature about variables related to resilience, focusing on analyzing the relations between resilience and personality traits in a sample of Spanish adults. Additionally, the role of hopelessness and age were examined on resilience capacity.

## METHOD

### Participants

The participants were a convenience sample comprising 439 Spanish adults (66.7% women; *M* = 43.73, *SD =* 26.41; age range = 18–98 years) recruited from public institutions: universities, universities for older adults, seniors' associations, and community centers. The participants were Spanish speakers and were of medium‐high socioeconomic status. The sample was divided into two groups: young adults (*n* = 227; 81.9% women; *M* = 19.86, *SD =* 1.30; age range = 18–24 years) and older adults (*n* = 212; 50.5% women; *M* = 71.37, *SD =* 8.76; age range 60–98 years). Inclusion criteria were: (1) being literate; (2) for the older group, having no cognitive difficulties. Cognitive impairment was tested using the Mini‐Mental Status Examination (Lobo *et al*., 2002); and (3) giving signed informed consent. Following these criteria, five participants were excluded for presenting scores compatible with cognitive impairment.

### Measures

#### Connor–Davidson resilience scale

To measure resilience, we administered the 10‐item Connor–Davidson Resilience Scale (CD‐RISC; Campbell‐Sills & Stein, 2007; Spanish adaptation by Notario‐Pacheco, Solera‐Martínez, Serrano‐Parra, Bartolomé‐Gutiérrez, García‐Campayo & Martínez‐Vizcaíno, 2011), which is scored on a Likert‐type scale with five options from *never* to *almost always*. The final score is the sum of all the items (range 0–40), where higher scores indicate higher levels of resilience. The Spanish version of the CD‐RISC has a Cronbach's alpha of 0.85.

#### NEO‐Five factor inventory

Neuroticism, extraversion, openness, agreeableness, and conscientiousness were assessed using the Spanish version of the NEO‐Five Factor Inventory (NEO‐FFI; Cordero, Pamos & Seisdedos, 2008). The NEO‐FFI comprises 60 items with five response options that range from strongly *disagree* to *strongly agree*. This instrument has shown an acceptable internal consistency, with a Cronbach's alpha of between 0.70 and 0.80.

#### Beck hopelessness scale

To measure future hopelessness, we used the Beck Hopelessness Scale (BHS; Beck, Weissman, Lester & Trexler, 1974; Spanish adaptation by Aguilar, Hidalgo, Cano, López, Campillo & Hernández, 1995), a unidimensional instrument comprising 22 dichotomous items, where higher scores indicate higher levels of hopelessness. The Spanish version of the BHS has shown good psychometric properties (Aguilar *et al*., 1995).

### Procedure

The study protocol was approved by the Clinical Research Ethics Committee of our autonomous community (Record No 6/2016). First, we presented the project to the management teams of the collaborating institutions to obtain the authorizations to implement the project. After approval, members of the research team visited all the different locations during in‐person activities in order to inform participants about the project. They explained the study aims, answered any questions and requested the participants' signed informed consent. Data were collected by four experimenters in a single session lasting around 60 min. The assessments were conducted in group format at the collaborating institutions, during the normal schedule.^1^

### Data analysis

Statistical analyses were conducted using IBM SPSS Statistics 24 (SPSS, Inc., Chicago, IL). The criterion for statistical significance was set at *p ≤* 0.05. Logical tests and range tests were conducted, as were data consistency tests. After reviewing and cleaning the data, we conducted exploratory analyses and other analyses to categorize and transform the variables. The specific data analysis plan included, first, a descriptive analysis of the main study variables and a *t‐*test for independent samples to determine any possible age‐related statistical differences. Second, to examine the association between resilience, personality traits and hopelessness, partial correlation coefficients were estimated, with adjustments made for age. Finally, hierarchical regression models were tested to determine in whole sample the variables that predicted resilience (i.e., age, personality traits, and hopelessness).

## RESULTS

### Descriptive results

The participants' characteristics are summarized in Table 1. Additionally, independent samples *t‐*tests were used to analyze possible statistical differences between age groups. Overall, the young group presented higher mean values in resilience, neuroticism, extraversion, and openness (*p* < 0.05), while the older group had higher mean values in hopelessness (*p <* 0.001). There were no statistically significant differences by age in personality traits, namely, agreeableness and conscientiousness.

**Table 1:** Characteristics of the study sample by age group

Variables	Total (*N* = 439)	Young group (*n* = 227)	Older group (*n* = 212)	*p* for difference	Cohen's *d*
Resilience	27.16 (6.63)	27.97 (5.69)	26.30 (7.42)	0.01	0.25
Personality traits					
Neuroticism	22.73 (7.61)	24.25 (7.95)	21.07 (6.88)	<0.001	0.43
Extraversion	29.78 (6.95)	31.36 (6.89)	28.08 (6.62)	<0.001	0.49
Openness	26.15 (6.64)	28.50 (6.08)	23.57 (6.28)	<0.001	0.79
Agreeableness	29.77 (5.62)	29.69 (5.02)	29.85 (6.21)	0.76	0.10
Conscientiousness	30.79 (6.06)	31.09 (5.98)	30.47 (6.16)	0.29	0.04
Hopelessness	5.03 (4.07)	3.73 (3.08)	6.43 (4.54)	<0.001	−0.70

*Note*: Resilience range: 0–40; Personality traits range: 0–48, for each factor; Hopelessness range: 0–20.

### Correlation results

After adjusting for age, partial correlation coefficients between personality traits and hopelessness and resilience showed that resilience was significantly related to all the personality traits (Table 2). Positive associations were found for extraversion, openness, agreeableness and conscientiousness, and a negative association was found between resilience and neuroticism. We also found that resilience was negatively and significantly related to hopelessness. In addition, as expected, while most personality traits were negatively associated with hopelessness, neuroticism showed a positive relation.

**Table 2:** Partial correlation coefficients among study variables, controlling for age

	1	2	3	4	5	6	7
1. Resilience	‐						
2. Neuroticism	−0.53**	‐					
3. Extraversion	0.43**	−0.40**	‐				
4.Openness	0.22**	−0.03	0.11*	‐			
5. Agreeableness	0.16**	−0.21**	0.14**	−0.03	‐		
6. Conscientiousness	0.27**	−0.29**	0.19**	0.04	0.32**	‐	
7. Hopelessness	−0.50**	0.49**	−0.32**	−0.13*	−0.17**	−0.31**	‐

*Note*: *N* = 439.

* *p <* 0.05.

** *p <* 0.001.

### Hierarchical regression analyses

For the whole study sample (*N* = 439), the hierarchical regression model was conducted with age, personality traits (i.e., neuroticism, extraversion, openness, agreeableness, and conscientiousness) and hopelessness as predictor variables, and resilience as dependent variable. Age was entered in the regression in Step 1, personality traits were entered in the following steps. In the final step, hopelessness scores were added (Table 3). The results showed the variables that explained resilience were neuroticism, extraversion, openness, and hopelessness. Interestingly, age was initially a predictor for resilience; however, when hopelessness was included in the regression model, age was no longer statistically significant.

**Table 3:** Hierarchical regression results for resilience

Dependent variable	Step	Predictor	*β*	*t*	*p*	Adjusted *R* ^*2*^	Δ*R* ^*2*^
Resilience	1	Age	−0.14	−2.85	0.005	0.02	0.02*
	2	Age	−0.24	−5.61	<0.0001	0.29	0.27**
		Neuroticism	−0.53	−12.35	<0.0001		
	3	Age	−0.17	−3.92	<0.0001	0.35	0.06**
		Neuroticism	−0.43	−9.46	<0.0001		
		Extraversion	0.27	5.95	<0.0001		
	4	Age	−0.10	−2.23	0.03	0.38	0.03**
		Neuroticism	−0.43	−9.74	<0.0001		
		Extraversion	0.25	5.61	<0.0001		
		Openness	0.19	4.35	<0.0001		
	5	Age	−0.10	−2.25	0.03	0.38	0.001
		Neuroticism	−0.42	−9.44	<0.0001		
		Extraversion	0.25	5.54	<0.0001		
		Openness	0.19	4.33	<0.0001		
		Agreeableness	0.04	0.85	0.39		
	6	Age	−0.10	−2.02	0.05	0.39	0.01*
		Neuroticism	−0.40	−8.80	<0.0001		
		Extraversion	0.24	5.37	<0.0001		
		Openness	0.19	4.33	<0.0001		
		Agreeableness	0.003	0.07	0.94		
		Conscientiousness	0.12	2.76	0.01		
	7	Age	0.003	0.06	0.95	0.43	0.04**
		Neuroticism	−0.30	−6.40	<0.0001		
		Extraversion	0.21	4.83	<0.0001		
		Openness	0.16	3.85	<0.0001		
		Agreeableness	−0.01	−0.02	0.98		
		Conscientiousness	0.08	1.86	0.06		
		Hopelessness	−0.26	5.47	<0.0001		

*Note*: The data are presented as standardized regression coefficients.

* *p <* 0.001.

** *p <* 0.0001.

## DISCUSSION

The main aim of this work was to analyze the association between resilience and personality traits in a sample of Spanish participants. Resilience is currently considered a complex construct made up of constitutional variables and aptitudes that facilitate overcoming life challenges. It is worth highlighting that a resilient personality is characterized by low neuroticism and relatively high levels of the other traits (Bohane, Maguire & Richardson, 2017). After adjusting for age, our results revealed a significant relation between personality traits and resilience levels, which is consistent with the findings of previous studies (e.g., Campbell‐Sills, Cohan & Stein, 2006; McDonnell & Semkovska, 2020; see Oshio *et al*., 2018, for a review). Specifically, resilience was negatively related to neuroticism, reinforcing the notion that vulnerability to stress is characteristic of this trait, with individuals scoring high in neuroticism being likely to also present lower resilience levels (Campbell‐Sills *et al*., 2006), which, in turn, might be predictors of mental health problems (McDonnell & Semkovska, 2020; Perna, Riva, Defillo, Sangiorgio, Nobile & Caldirola, 2020). In addition, we found that resilience was directly associated with extraversion, openness, agreeableness, and conscientiousness. Various studies have reported similar findings (Davey, Eaker & Walters, 2016; Färber & Rosendahl, 2020; McDonnell & Semkovska, 2020; Oshio *et al*., 2018), which would underscore the important role of resilience as an effective mechanism for dealing with adversity (Oshio *et al*., 2018).

As regards the role of hopelessness, it has been reported that negative future‐oriented thinking has a detrimental impact on resilience (Gooding, Hurst, Johnson & Tarrier, 2012) and has been shown to be a factor predicting lower levels of resilience (Hjemdal *et al*., 2012). In line with previous findings (Gooding *et al*., 2012; MacLeod, Musich, Hawkins, Alsgaard & Wicker, 2016), our data show an inverse relation between resilience and hopelessness, with hopelessness being a significant predictor of lower levels of resilience. Additionally, the traits of neuroticism and introversion increase the likelihood of experiencing hopelessness (Hjemdal *et al*., 2012), a finding which is consistent with our results. Concerning age, the levels of hopelessness found in our study were significantly higher in older adults than in their young counterparts. Age differences in hopelessness may underlie the changes and losses experienced over life, arguably promoting a pessimistic outlook on the future. Aging for example, is a life stage with greater exposure to health problems, disability, death, loneliness, and pain (Harithasan, Mukari, Ishak, Shahar & Yeong, 2020; Meichsner, O'Connor, Skritskaya & Shear, 2020), while young adulthood is considered a period of change in which identity is consolidated and decisions are made related to personal and professional life (Wolf & Zimprich, 2015). It is worth noting, however, that most older people do not experience hopelessness, but when it appears, it differs to that experienced by young adults, since certain elements of the aging process generate uncertainty and negative expectations (see Hernandez & Overholser, 2020, for a review).

As for age differences, these appear not to have been a core focus of studies on resilience, and conclusions in this area thus remain uncertain (Lee *et al*., 2013). In studies using the CD‐RISC, for example, no significant relations have been found between age and levels of resilience, suggesting a more specific role of life experiences (Pulido *et al*., 2020). However, when other measures have been used, older adults have been shown to be more resilient with respect to emotional regulation and problem solving, while resilience in younger adults is related to social support (Gooding *et al*., 2012). On the other hand, MacLeod *et al*. (2016) indicated that higher levels of resilience were associated with increasing age, suggesting that young adults are not always as resilient as older ones. Our results showed that the young adults were more resilient than their older counterparts, although age was not a significant predictor of resilience. It is important to highlight that neuroticism, extraversion, openness, and hopelessness were the variables that explained the levels of resilience. Therefore, the effect of these variables on the levels of resilience seems important in different generations. Consequently, along with the demonstrated relation between resilience and personality traits (Oshio *et al*., 2018), the role of hopelessness is also highlighted. However, to our understanding, this is a topic that has been insufficiently explored and our approaches could be considered preliminary results, with the purpose of subsequently conducting a more exhaustive study on resilience and hopelessness. It might even be considered a transdiagnostic variable, due to its impact on mental health, because it is a construct that reflects negative evaluations about the future and is also a significant predictor of suicidal behaviors and ideation (e.g., Hernandez & Overholser, 2020; Hirsch, Hall, Wise, Brooks, Chang & Sirois, 2019; Sueki, 2022).

The findings from the present work need to be interpreted in light of the following limitations. First, the cross‐sectional study nature prevents us from making cause‐effect inferences. Longitudinal data would yield more complete information on within‐participant development in each of the variables analyzed, considering that the ways in which resilience manifests itself may change for different types of adversity, and resilience can be understood as the result of the experiential learning and adaptation over time (Cosco, Kok, Wister & Howse, 2019; Malhi *et al*., 2019). Second, regarding the resilience measure, it is necessary to be cautious when generalizing results in this area, because different scales incorporate different constructs, due to the diversity in the operation of resilience (Sarubin *et al*., 2015). In fact, there are different approaches about resilience being a process or an individual disposition, or both (Leys, Arnal, Wollast, Rolin, Kotsou, & Fossion, 2020). We have used the CD‐RISC scale, which conceptualizes resilience as the personal qualities that enable one to thrive in the face of adversity (Connor & Davidson, 2003), even though, we could incorporate different resilience measures in future studies. Likewise, we could also evaluate stress coping strategies that complement the information provided by resilience when comparing age groups (e.g., Nieto, Romero, Ros *et al*., 2020), following the approaches of the Socioemotional Selectivity Theory (Carstensen, 2006), which proposes the shift of personal goals and behaviors with age. In the same vein, it has also been suggested that age strengthens the ability to distance oneself from stressful situations and reassess them positively (Schryer & Ross, 2012). Finally, regarding the external and cultural validity of the results, our data were collected from a convenience sample in which all participants were Caucasian Spanish adults from a medium‐high economic status background. We consider that future studies should focus on the replication of our findings in samples from different cultures and socioeconomic statuses with different levels of risk exposure. For example, Blessin, Lehmann, Kunzler, van Dick and Lieb (2022) suggest that because the world is increasingly intercultural, we should reassess the way we consider different countries in psychological research, which could help to complete our knowledge about effective resilience approaches and related factors.

To sum up, resilience was related to personality traits and hopelessness. In addition, young adults exhibited higher levels of resilience than older adults. However, age was not a significant predictor of resilience. Importantly, neuroticism, extraversion, openness, and hopelessness explained resilience levels in our study sample. These findings could be interesting to improve mental health interventions, due to the recognized importance of resilience in competently overcoming adverse life circumstances. Additionally, these approaches may also be applicable in the global COVID‐19 pandemic situation, as there is great concern about the psychological impacts of the pandemic on mental health. Therefore, enhancing resilience might have positive implications in terms of mental health status (Tseliou & Ashfield‐Watt, 2022; Verdolini, Amoretti, Montejo *et al*., 2021).

Data are available on request from the authors. The data that support the findings of this study are available from the corresponding author upon reasonable request.

Castilla‐La Mancha Department of Education, Culture and Sports and the European Regional Development. SPBLY/19/180501/000181.

## Data Availability

Availability of data Data are available on request from the authors. The data that support the findings of this study are available from the corresponding author upon reasonable request.

## Endnote

^1^
All data were collected in person before the COVID‐19 pandemic.

## References

[sjop12866-bib-0001] Aguilar, E.J. , Hidalgo, M.D. , Cano, R. , López, J.C. , Campillo, M. & Hernández, M. (1995). Estudio prospectivo de la desesperanza en pacientes psicóticos: Características psicométricas de la Escala de Desesperanza de Beck. Anales de Psiquiatría, 11, 121–125.

[sjop12866-bib-0002] Alessandri, G. , Vecchione, M. , Donnellan, B.M. , Eisenberg, N. , Caprara, G.V. & Cieciuch, J. (2014). On the cross‐cultural replicability of the resilient, undercontrolled, and overcontrolled personality types. Journal of Personality, 82, 340–353. 23957593 10.1111/jopy.12065

[sjop12866-bib-0003] Barton, G. , McKay, L. , Garvis, S. & Sappa, V. (2020). Introduction: Defining and theorizing key concepts of resilience and well‐being and arts‐based research. In L. McKay , G. Barton , S. Garvis & V. Sappa (Eds.), Arts‐based research, resilience and well‐being across the lifespan (pp. 1–12). Springer International Publishing. 10.1007/978-3-030-26053-8_18

[sjop12866-bib-0004] Beck, A.T. , Weissman, A. , Lester, D. & Trexler, L. (1974). The measurement of pessimism: The hopelessness scale. Journal of Consulting and Clinical Psychology, 42, 861–865. 4436473 10.1037/h0037562

[sjop12866-bib-0005] Beutel, M.E. , Glaesmer, H. , Decker, O. , Fischbeck, S. & Brähler, E. (2009). Life satisfaction, distress, and resiliency across the life span of women. Menopause, 16, 1132–1138. 19543128 10.1097/gme.0b013e3181a857f8

[sjop12866-bib-0006] Biggs, A. , Brough, P. & Drummond, S. (2017). Lazarus and Folkman's psychological stress and coping theory. In C.L. Cooper & J.C. Quick (Eds.), The handbook of stress and health: A guide to research and practice (pp. 351–364). Chinchester: Wiley Blackwell.

[sjop12866-bib-0007] Blessin, M. , Lehmann, S. , Kunzler, A. M. , van Dick, R. & Lieb, K. (2022). Resilience interventions conducted in Western and eastern countries: A systematic review. International Journal of Environmental Research and Public Health, 19(11), 6913. 10.3390/ijerph19116913 35682495 PMC9180776

[sjop12866-bib-0008] Bohane, L. , Maguire, N. & Richardson, T. (2017). Resilients, overcontrollers and undercontrollers: A systematic review of the utility of a personality typology method in understanding adult mental health problems. Clinical Psychology Review, 57, 75–92. 28850932 10.1016/j.cpr.2017.07.005

[sjop12866-bib-0009] Bonanno, G.A. , Westphal, M. & Mancini, A.D. (2011). Resilience to loss and potential trauma. Annual Review of Clinical Psychology, 7, 511–535. 10.1146/annurev-clinpsy-032210-104526 21091190

[sjop12866-bib-0010] Bryant, R.A. , Schnurr, P.P. & Pedlar, D. (2022). Addressing the mental health needs of civilian combatants in Ukraine. The Lancet Psychiatry, 9(5), 346–347. 35305300 10.1016/S2215-0366(22)00097-9

[sjop12866-bib-0011] Campbell‐Sills, L. , Cohan, S.L. & Stein, M.B. (2006). Relation of resilience to personality, coping, and psychiatric symptoms in young adults. Behaviour Research and Therapy, 44, 585–599. 15998508 10.1016/j.brat.2005.05.001

[sjop12866-bib-0012] Campbell‐Sills, L. , Forde, D.R. & Stein, M.B. (2009). Demographic and childhood environmental predictors of resilience in a community sample. Journal of Psychiatric Research, 43, 1007–1012. 19264325 10.1016/j.jpsychires.2009.01.013

[sjop12866-bib-0013] Campbell‐Sills, L. & Stein, M.B. (2007). Psychometric analysis and refinement of the Connor‐Davidson resilience scale (CD‐RISC): Validation of a 10‐item measure of resilience. Journal of Traumatic Stress, 20, 1019–1028. 18157881 10.1002/jts.20271

[sjop12866-bib-0014] Carstensen, L.L. (2006). The influence of a sense of time on human development. Science, 312, 1913–1915. 16809530 10.1126/science.1127488 PMC2790864

[sjop12866-bib-0015] Chioqueta, A.P. & Stiles, T.C. (2005). Personality traits and the development of depression, hopelessness, and suicide ideation. Personality and Individual Differences, 38, 1283–1291.

[sjop12866-bib-0016] Cloninger, C.R. & Zohar, A.H. (2011). Personality and the perception of health and happiness. Journal of Affective Disorders, 128, 24–32. 20580435 10.1016/j.jad.2010.06.012

[sjop12866-bib-0017] Connor, K.M. & Davidson, J.R.T. (2003). Development of a new resilience scale: The Connor‐Davidson resilience scale (CD‐RISC). Depression and Anxiety, 18, 76–82. 12964174 10.1002/da.10113

[sjop12866-bib-0018] Cordero, A. , Pamos, A. & Seisdedos, N. (2008). Inventario NEO Reducido de Cinco Factores (NEO‐FFI). Madrid: TEA Ediciones.

[sjop12866-bib-0019] Cosco, T.D. , Kok, A. , Wister, A. & Howse, K. (2019). Conceptualising and operationalising resilience in older adults. Health Psychology and Behavioral Medicine, 7, 90–104. 34040841 10.1080/21642850.2019.1593845 PMC8114384

[sjop12866-bib-0020] Costa, P.T. & McCrae, R.R. (1992). Normal personality assessment in clinical practice: The NEO personality inventory. Psychological Assessment, 4, 5–13.

[sjop12866-bib-0021] Costa, P.T. , McCrae, R.R. & Löckenhoff, C.E. (2019). Personality across the life span. Annual Review of Psychology, 70, 423–448. 10.1146/annurev-psych-010418-103244 30231002

[sjop12866-bib-0022] Davey, M. , Eaker, D.G. & Walters, L.H. (2016). Resilience processes in adolescents: Personality profiles, self‐worth, and coping. Journal of Adolescent Research, 18, 347–362.

[sjop12866-bib-0023] Färber, F. & Rosendahl, J. (2020). Trait resilience and mental health in older adults: A meta‐analytic review. Personality and Mental Health, 14, 361–375. 32573068 10.1002/pmh.1490

[sjop12866-bib-0024] Gillespie, B.M. , Chaboyer, W. & Wallis, M. (2009). The influence of personal characteristics on the resilience of operating room nurses: A predictor study. International Journal of Nursing Studies, 46, 968–976. 17915223 10.1016/j.ijnurstu.2007.08.006

[sjop12866-bib-0025] Gooding, P.A. , Hurst, A. , Johnson, J. & Tarrier, N. (2012). Psychological resilience in young and older adults. International Journal of Geriatric Psychiatry, 27, 262–270. 21472780 10.1002/gps.2712

[sjop12866-bib-0026] Grav, S. , Stordal, E. , Romild, U.K. & Hellzen, O. (2012). The relation among neuroticism, extraversion, and depression in the HUNT study: In relation to age and gender. Issues in Mental Health Nursing, 33, 777–785. 23146012 10.3109/01612840.2012.713082

[sjop12866-bib-0027] Harithasan, D. , Mukari, S.Z.‐M.S. , Ishak, W.S. , Shahar, S. & Yeong, W.L. (2020). The impact of sensory impairment on cognitive performance, quality of life, depression, and loneliness in older adults. International Journal of Geriatric Psychiatry, 35, 358–364. 31736109 10.1002/gps.5237

[sjop12866-bib-0028] Harnett, N.G. , van Rooij, S.J.H. , Ely, T.D. , Lebois, L.A.M. , Murty, V.P. , Jovanovic, T. *et al*. (2021). Prognostic neuroimaging biomarkers of trauma‐related psychopathology: Resting‐state fMRI shortly after trauma predicts future PTSD and depression symptoms in the AURORA study. Neuropsychopharmacology, 46, 1263–1271. 33479509 10.1038/s41386-020-00946-8 PMC8134491

[sjop12866-bib-0029] Hernandez, S.C. & Overholser, J.C. (2020). A systematic review of interventions for hope/hopelessness in older adults. Clinical Gerontologist, 8, 1–15. 10.1080/07317115.2019.1711281 31913808

[sjop12866-bib-0030] Hirsch, J.K. , Hall, B.B. , Wise, H.A. , Brooks, B.D. , Chang, E.C. & Sirois, F.M. (2019). Negative life events and suicide risk in college students: Conditional indirect effects of hopelessness and self‐compassion. Journal of American College Health, 25, 1–8. 10.1080/07448481.2019.1692023 31765290

[sjop12866-bib-0031] Hjemdal, O. , Friborg, O. & Stiles, T.C. (2012). Resilience is a good predictor of hopelessness even after accounting for stressful life events, mood, and personality (NEO‐PI‐R). Scandinavian Journal of Psychology, 53, 174–180. 22092028 10.1111/j.1467-9450.2011.00928.x

[sjop12866-bib-0032] Kalisch, R. , Baker, D.G. , Basten, U. , Boks, M.P. , Bonanno, G.A. , Brummelman, E. *et al*. (2017). The resilience framework as a strategy to combat stress‐related disorders. Nature Human Behaviour, 1, 784–790. 10.1038/s41562-017-0200-8 31024125

[sjop12866-bib-0033] Lamond, A.J. , Depp, C. , Allison, M. , Langer, R. , Reichstadt, J. , Moore, D.J. *et al*. (2008). Measurement and predictors of resilience among community‐dwelling older women. Journal of Psychiatric Research, 43, 148–154. 18455190 10.1016/j.jpsychires.2008.03.007 PMC2613196

[sjop12866-bib-0034] Lee, J.H. , Nam, S.K. , Kim, A.‐R. , Kim, B. , Lee, M.Y. & Lee, S.M. (2013). Resilience: A meta‐analytic approach. Journal of Counseling & Development, 91, 269–279.

[sjop12866-bib-0035] Leys, C. , Arnal, C. , Wollast, R. , Rolin, H. , Kotsou, I. & Fossion, P. (2020). Perspectives on resilience: Personality trait or skill? European Journal of Trauma & Dissociation, 4, 100074.

[sjop12866-bib-0068] Lobo, A. , Saz, P. , Marcos, G. & Grupo de Trabajo, Z. (2002). MMSE: Examen cognoscitivo mini‐mental. Madrid: Tea Ediciones.

[sjop12866-bib-0036] Lü, W. , Wang, Z. , Liu, Y. & Zhang, H. (2014). Resilience as a mediator between extraversion, neuroticism and happiness, PA and NA. Personality and Individual Differences, 63, 128–133.

[sjop12866-bib-0037] MacLeod, S. , Musich, S. , Hawkins, K. , Alsgaard, K. & Wicker, E.R. (2016). The impact of resilience among older adults. Geriatric Nursing, 37, 266–272. 27055911 10.1016/j.gerinurse.2016.02.014

[sjop12866-bib-0038] Malhi, G.S. , Das, P. , Bell, E. , Mattingly, G. & Mannie, Z. (2019). Modelling resilience in adolescence and adversity: A novel framework to inform research and practice. Translational Psychiatry, 9, 1–16. 31772187 10.1038/s41398-019-0651-y PMC6879584

[sjop12866-bib-0039] Masten, A.S. (2015). Pathways to integrated resilience science. Psychological Inquiry, 26, 187–196.

[sjop12866-bib-0040] McDonnell, S. & Semkovska, M. (2020). Resilience as mediator between extraversion, neuroticism, and depressive symptoms in university students. Journal of Positive Psychology and Wellbeing, 4, 1–16.

[sjop12866-bib-0041] McGinnis, D. (2018). Resilience, life events, and well‐being during midlife: Examining resilience subgroups. Journal of Adult Development, 25, 198–221. 30174384 10.1007/s10804-018-9288-y PMC6105201

[sjop12866-bib-0042] Meichsner, F. , O'Connor, M. , Skritskaya, N. & Shear, M.K. (2020). Grief before and after bereavement in the elderly: An approach to care. The American Journal of Geriatric Psychiatry, 28, 560–569. 32037292 10.1016/j.jagp.2019.12.010

[sjop12866-bib-0043] Murphy, L.B. (1987). Further reflections on resilience. In E.J. Anthony & B.J. Cohler (Eds.), The invulnerable child (pp. 84–105). New York: Guilford Press.

[sjop12866-bib-0044] Nieto, M. , Romero, D. , Ros, L. , Zabala, C. , Martínez, M. , Ricarte, J.J. *et al*. (2020). Differences in coping strategies between young and older adults: The role of executive functions. International Journal of Aging & Human Development, 90, 28–49. 30612437 10.1177/0091415018822040

[sjop12866-bib-0045] Notario‐Pacheco, B. , Solera‐Martínez, M. , Serrano‐Parra, M.D. , Bartolomé‐Gutiérrez, R. , García‐Campayo, J. & Martínez‐Vizcaíno, V. (2011). Reliability and validity of the Spanish version of the 10‐item Connor‐Davidson resilience scale (10‐item CD‐RISC) in young adults. Health and Quality of Life Outcomes, 9, 63. 21819555 10.1186/1477-7525-9-63 PMC3173284

[sjop12866-bib-0046] Ogle, C.M. , Rubin, D.C. & Siegler, I.C. (2014). Changes in neuroticism following trauma exposure. Journal of Personality, 82, 93–102. 23550961 10.1111/jopy.12037 PMC3926905

[sjop12866-bib-0047] Ong, A.D. , Bergeman, C.S. , Bisconti, T.L. & Wallace, K.A. (2006). Psychological resilience, positive emotions, and successful adaptation to stress in later life. Journal of Personality and Social Psychology, 91, 730–749. 17014296 10.1037/0022-3514.91.4.730

[sjop12866-bib-0048] Oshio, A. , Kaneko, H. , Nagamine, S. & Nakaya, M. (2003). Construct validity of the adolescent resilience scale. Psychological Reports, 93, 1217–1222. 14765593 10.2466/pr0.2003.93.3f.1217

[sjop12866-bib-0049] Oshio, A. , Taku, K. , Hirano, M. & Saeed, G. (2018). Resilience and big five personality traits: A meta‐analysis. Personality and Individual Differences, 127, 54–60.

[sjop12866-bib-0050] Perna, G. , Riva, A. , Defillo, A. , Sangiorgio, E. , Nobile, M. & Caldirola, D. (2020). Heart rate variability: Can it serve as a marker of mental health resilience? Journal of Affective Disorders, 263, 754–761. 31630828 10.1016/j.jad.2019.10.017

[sjop12866-bib-0051] Pulido, M. , Fernández, M.D. & Lopez, E. (2020). Measurement invariance across gender and age in the Connor‐Davidson resilience scale (CD‐RISC) in a Spanish general population. Quality of Life Research: An International Journal of Quality‐of‐Life Aspects of Treatment, Care and Rehabilitation, 29, 1373–1384. 31845166 10.1007/s11136-019-02389-1

[sjop12866-bib-0052] Rodríguez, R. , Alonso, J. & Hernansaiz, H. (2016). Reliability and validity of the brief resilience scale (BRS) Spanish version. Psychological Assessment, 28, e101–e110. 26502199 10.1037/pas0000191

[sjop12866-bib-0053] Salisu, I. & Hashim, N. (2017). A critical review of scales used in resilience research. Journal of Business and Management, 19, 23–33.

[sjop12866-bib-0054] Sarubin, N. , Wolf, M. , Giegling, I. , Hilbert, S. , Naumann, F. , Gutt, D. *et al*. (2015). Neuroticism and extraversion as mediators between positive/negative life events and resilience. Personality and Individual Differences, 82, 193–198.

[sjop12866-bib-0055] Schneider, T.R. , Rench, T.A. , Lyons, J.B. & Riffle, R.R. (2012). The influence of neuroticism, extraversion and openness on stress responses. Stress and Health: Journal of the International Society for the Investigation of Stress, 28, 102–110. 22281953 10.1002/smi.1409

[sjop12866-bib-0056] Schryer, E. & Ross, M. (2012). Evaluating the valence of remembered events: The importance of age and self‐relevance. Psychology and Aging, 27, 237–242. 21463052 10.1037/a0023283

[sjop12866-bib-0057] Southwick, S.M. , Bonanno, G.A. , Masten, A.S. , Panter‐Brick, C. & Yehuda, R. (2014). Resilience definitions, theory, and challenges: Interdisciplinary perspectives. European Journal of Psychotraumatology, 5, 25338. 10.3402/ejpt.v5.25338 PMC4185134 25317257

[sjop12866-bib-0058] Sueki, H. (2022). Relationship between Beck hopelessness scale and suicidal ideation: A short‐term longitudinal study. Death Studies, 46, 467–472. 32180536 10.1080/07481187.2020.1740833

[sjop12866-bib-0059] Tseliou, F. & Ashfield‐Watt, P. (2022). The association between resilience resources, contextual factors and mental health status: A national population‐based study. BMC Public Health, 22, 1–13. 35351041 10.1186/s12889-022-13013-2 PMC8962564

[sjop12866-bib-0060] Ungar, M. & Theron, L. (2019). Resilience and mental health: How multisystemic processes contribute to positive outcomes. The Lancet Psychiatry, 7, 441–448. 31806473 10.1016/S2215-0366(19)30434-1

[sjop12866-bib-0061] Verdolini, N. , Amoretti, S. , Montejo, L. , García‐Rizo, C. , Hogg, B. , Mezquida, G. *et al*. (2021). Resilience and mental health during the COVID‐19 pandemic. Journal of Affective Disorders, 283, 156–164. 33556749 10.1016/j.jad.2021.01.055 PMC7845537

[sjop12866-bib-0062] Wagner, J. , Lüdtke, O. & Robitzsch, A. (2019). Does personality become more stable with age? Disentangling state and trait effects for the big five across the life span using local structural equation modeling. Journal of Personality and Social Psychology, 116, 666–680. 30714756 10.1037/pspp0000203

[sjop12866-bib-0063] Willey, B. , Mimmack, K. , Gagliardi, G. , Dossett, M.L. , Wang, S. , Udeogu, O.J. *et al*. (2022). Racial and socioeconomic status differences in stress, posttraumatic growth, and mental health in an older adult cohort during the COVID‐19 pandemic. The Lancet, 45, 101343. 10.1016/j.eclinm.2022.101343 PMC8917957 35291556

[sjop12866-bib-0064] Wolf, T. & Zimprich, D. (2015). Differences in the use of autobiographical memory across the adult lifespan. Memory, 23, 1238–1254. 25343565 10.1080/09658211.2014.971815

[sjop12866-bib-0065] World Health Organization . (2019). Ten facts on mental health. Retrieved from https://www.who.int/news‐room/facts‐in‐pictures/detail/mental‐health [Accessed 1st January 2022].

[sjop12866-bib-0066] World Health Organization . (2022). Mental disorders. Retrieved from https://www.who.int/news‐room/fact‐sheets/detail/mental‐disorders [Accessed 15th August 2022].

[sjop12866-bib-0067] Zhu, Y. , Zhang, L. , Zhou, X. , Li, C. & Yang, D. (2021). The impact of social distancing during COVID‐19: A conditional process model of negative emotions, alienation, affective disorders, and post‐traumatic stress disorder. Journal of Affective Disorders, 281, 131–137. 33316718 10.1016/j.jad.2020.12.004 PMC7723399

